# Development and validation of a prediction model for active tuberculosis case finding among HIV-negative/unknown populations

**DOI:** 10.1038/s41598-019-42372-x

**Published:** 2019-04-16

**Authors:** Yun-Ju Shih, Helen Ayles, Knut Lönnroth, Mareli Claassens, Hsien-Ho Lin

**Affiliations:** 1 Institute of Epidemiology and Preventive Medicine, National Taiwan University, Taipei, Taiwan; 2 Department of Clinical Research, London School of Hygiene and Tropical Medicine, London, UK; 3 Department of Public Health Sciences, Karolinska Institutet, Stockholm, Sweden; 4 Desmond Tutu Tuberculosis Centre, Department of Paediatrics and Child Health, Stellenbosch University, Cape Town, South Africa

## Abstract

A prediction model of prevalent pulmonary tuberculosis (TB) in HIV negative/unknown individuals was developed to assist systematic screening. Data from a large TB screening trial were used. A multivariable logistic regression model was developed in the South African (SA) training dataset, using TB symptoms and risk factors as predictors. The model was converted into a scoring system for risk stratification and was evaluated in separate SA and Zambian validation datasets. The number of TB cases were 355, 176, and 107 in the SA training, SA validation, and Zambian validation datasets respectively. The area under curve (AUC) of the scoring system was 0·68 (95% CI 0·64-0·72) in the SA validation set, compared to prolonged cough (0·58, 95% CI 0·54-0·62) and any TB symptoms (0·6, 95% CI 0·56–0·64). In the Zambian dataset the AUC of the scoring system was 0·66 (95% CI 0·60–0·72). In the cost-effectiveness analysis, the scoring system dominated the conventional strategies. The cost per TB case detected ranged from 429 to 1,848 USD in the SA validation set and from 171 to 10,518 USD in the Zambian dataset. The scoring system may help targeted TB case finding under budget constraints.

## Introduction

Tuberculosis (TB) continues to be highly prevalent in developing countries where the resources for TB screening and diagnosis are limited^1,2^. In order to interrupt the chain of transmission, a TB control and prevention system must include prompt diagnosis and treatment. However, findings from TB prevalence surveys reveal a high burden of undetected TB cases even in the presence of quality-assured programmatic TB diagnosis and treatment, suggesting that TB patients tend to undergo a long and sometimes unsuccessful journey of health care seeking^3^. One way of addressing the challenge of delayed and undiagnosed TB is to perform active case finding activities where TB cases are actively and systematically sought in the community^4^.

Under the resource constraints, systematic screening for active TB usually starts with initial screening using TB symptoms, followed by confirmatory diagnostic processes for those who are symptom positive. One method proposed by the World Health Organization (WHO) is to use prolonged cough as the initial screening rule while acknowledging its low sensitivity. The sensitivity and specificity of prolonged cough were 35% (95% CI 24–46%) and 95% (95% CI 93–97%) respectively in the general population regardless of human immunodeficiency virus (HIV) status^5^. An alternative method is to use the presence of any TB symptom as the initial rule, which showed a corresponding sensitivity and specificity of 77% (95% CI 68–86%) and 68% (95%CI 50–85%) respectively in the general population regardless of HIV status^5^. These two methods represent two extremes on the TB screening continuum, where either too many TB-negative individuals receive further testing (in the case of any TB symptom) or too few TB-positive cases are included (in the case of prolonged cough). This trade-off between sensitivity and specificity presents a challenge to the selection of an appropriate screening strategy especially if there is a budget constraint.

In addition to conventional screening strategies, prediction models have been increasingly used to assist development of new screening approaches^6^. We hypothesized that prediction models that include TB risk factors in addition to symptoms may have improved prediction accuracy than conventional methods. We also hypothesized that a prediction model can stratify people into different risk groups and provide the basis of cost-effective screening options. We developed and validated such a prediction model, aiming to improve prediction accuracy and to assist the implementation of systematic screening in the community for prevalent TB. We focused the present analysis on TB screening among individuals with negative or unknown HIV status in the community, as the screening algorithm for people living with HIV has been previously described^7^.

## Methods

### Study design and participants

The study population consisted of participants in a TB prevalence survey of the Zambia South Africa Tuberculosis and HIV/AIDS Reduction (ZAMSTAR) trial conducted from January to December 2010. A prevalence survey was done to measure TB prevalence (the primary outcome of the trial) and was described elsewhere^8,9^. The dataset contained information from participants from eight communities in South Africa (SA) and 16 communities in Zambia. Participants who were receiving TB treatment at the time of data collection were excluded to avoid bias from the effect of TB treatment on TB symptoms. Only participants with HIV negative or HIV unknown status were included in this study since HIV positive participants might present different TB symptoms and signs compared to HIV negative individuals^10^. Participants without information on previous TB history were excluded since we were interested in this factor as a predictor. Overall 26,864 individuals from SA and 29,381 from Zambia were included in the analysis (Appendix Fig. 1). The SA dataset was further separated into a training and a validation set. To meet the requirement of more than 100 cases in a validation set^11^, the SA dataset was randomly split into two thirds (355 cases among 17,912 participants) for training and one third (176 cases among 8,952 participants) for (internal) validation. The Zambian dataset (107 cases in 29,381 participants) was used as the second (external) validation set to evaluate the performance of the SA model when extrapolated to a different epidemiological setting with lower TB prevalence.

### Measurement of outcome and predictors

The outcome of the study was prevalent active culture-confirmed TB. All ZAMSTAR participants were required to produce one sputum sample at the time of interview for *Mycobacterium tuberculosis* culture and speciation regardless of TB symptoms. The laboratory algorithm has been described elsewhere^3,12^. The results of the culture examination were used as the gold standard for diagnosing prevalent TB in the prediction model. Symptoms and risk factors of TB were considered as candidate predictor variables and the information was based on self-report only. TB-related symptoms included: cough less than two weeks (cough), cough more than two weeks (prolonged cough), fever, weight loss, night sweats, chest pain, shortness of breath, and sputum production. The second group of variables was well described risk factors of TB, including age, sex, ever smoking, ever drink alcohol, body mass index (BMI), and known diabetes mellitus (DM)^13–18^. Self-reported household TB history and personal TB history were also included as potential predictors. The two types of TB history were treated as mutually exclusive states as they reflected different levels of prior TB exposure. If an individual had a personal TB history and a household TB history, the person was classified as having a personal TB history since it is a stronger indication of TB exposure.

### Statistical analysis

Multivariable logistic regression was used to estimate the relationship (odds ratio, OR) between multiple predictors and prevalent TB in the SA training dataset. Model selection was done using backward elimination based on the Akaike information criterion (AIC). The results of the final model were converted into a scoring system by dividing the coefficients by the smallest coefficient value in the results of final regression model^19^. The discrimination of the scoring system was showed in Receiver Operating Characteristic (ROC) curves. The area under the ROC curve (AUC) was calculated to compare the prediction performance of the newly developed model against prolonged cough or any TB symptom.

In addition to AUC, cost-effectiveness analyses were conducted to compare systematic screening algorithms that applied the different initial screening methods, namely the scoring system, prolonged cough, and any TB symptom, in the validation datasets. The algorithms represent possible systematic screening strategies in the community in the context of the healthcare systems in SA and Zambia (Appendix Fig. 2). For each algorithm the expected yield of TB cases and associated costs were calculated in the two validation datasets. The cost items included the costing from the confirmatory tests. The staff time and the patient costs were not included in this study because they might vary in different settings. (Appendix Table 1). The incremental cost-effectiveness ratio (ICER) and the average cost-effectiveness ratio (ACER) was calculated for each algorithm using different initial screening methods. All analyses were done under 1000 bootstrapped samples. Lastly, the median value of each screening algorithm from bootstrapping results was applied to several hypothetical populations with different TB prevalences to calculate the number of TB cases detected and associated costs at different cutoff values of the scoring system.

In the main analysis, BMI was not included as a potential predictor due to the high proportion of missing values (66%) in the SA dataset. Cumulating evidence suggests that BMI is strongly associated with the risk of TB^17^. Therefore, a sensitivity analysis was conducted using multiple imputation to account for missing BMI values. The missing values were imputed for ten times in the training dataset and two testing datasets were performed independently. Rubin’s rule was adopted to pool the results of regression models and the model performances^20^. The final prediction model in the sensitivity analysis included the predictors which were selected in more than half of the ten imputed training datasets^21^. The pooled sensitivities and specificities of the prediction models were applied to the cost-effectiveness analysis to estimate the benefit and cost in the two validation datasets.

### Ethics approval and consent to participate

The original study (ZAMSTAR) had the ethics approval from University of Zambia, Stellenbosch University and the London School of Hygiene and Tropical Medicine. All the individuals participated in the study had provided written informed consent. This analysis had additional ethics approval from Stellenbosch University (S14/09/178). All methods used in this study were performed in accordance with the relevant guidelines and regulations.

## Results

The mean age of participants was over 30 years, and the majority (>60%) were female (Table 1). The proportion of unknown HIV status was 42.9% and 18.5% in the SA and Zambian participants respectively. Up to 35% and 24% of the SA and Zambian participants reported any TB-related symptoms (cough, weight loss, night sweats, and fever). The prevalence of TB was 2·0% and 0·36% among the SA and Zambian participants respectively. Among prevalent TB cases, the probabilities of presenting symptoms were higher than non-TB participants in both countries. The mean BMI of the TB positive individuals were lower than those TB negative individuals. Participants from South Africa had higher probabilities of known personal and household TB history than those who were from Zambia (Table 1).

**Table 1:** Descriptive statistics in the two datasets used in the analysis.

	South African dataset		Zambian dataset	
	TB (n = 531)	No TB (n = 26,343)	TB (n = 107)	No TB (n = 29,275)
Female	260 (49·0%)	16,047 (60·9%)	53 (49·5%)	18,948 (64·7%)
Age, mean (SD)	37·3 (SD: 15·2) (missing: 18)	34·2 (SD: 14·0)	31·3 (SD: 12·9) (missing:18)	32·7 (SD: 14·8) (missing: 388)
BMI, mean (SD)	23·0 (SD: 5·3) (missing: 356)	27·1 (SD: 6·7) (missing: 17,428)	21·0 (SD: 3·4) (missing: 11)	23·0 (SD: 4·5) (missing: 2,791)
Ever-smoker	208 (39·2%)	6,544 (24·4%)	32 (29·9%)	3,738 (12·8%)
Ever-drinker	322 (60·6%)	11,851 (44·1%)	66 (61·7%)	12,556 (42·9%)
Cough Sputum	132 (24·9%)	2,454 (9·3%)	31 (29·0%)	1,964 (6·7%)
No cough	366 (68·9%)	23,251 (88·3%)	71 (66·4%)	26,245 (89·6%)
Cough <2weeks	63 (11·9%)	1,840 (7·0%)	19 (17·8%)	2,224 (7·6%)
Cough > = 2weeks	102 (19·2%)	1,252 (4·8%)	17 (15·9%)	806 (2·8%)
Fever	133 (25·0%)	4,707 (17·5%)	14 (13·1%)	1,389 (4·7%)
Night sweats	157 (29·6%)	4,011 (14·9%)	16 (15·0%)	1,481 (5·1%)
Weight loss	146 (27·5%)	3,433 (12·8%)	18 (16·8%)	3,392 (11·6%)
Chest pain	94 (17·7%)	2,544 (9·5%)	22 (20·6%)	2,246 (7·7%)
Hard breath	81 (15·3%)	2,073 (7·7%)	18 (16·8%)	1,539 (5·3%)
Ever diagnosed with DM	40 (7·5%)	2,030 (7·6%)	4 (3·7%)	532 (1·8%)
Ever been on TB treatment	95 (17·9%)	2,686 (10·0%) (missing: 10)	9 (8·4%)	990 (3·4%) (missing: 1)
Household TB history	78 (14·7%)	2,896 (10·8%)	3 (2·8%)	909 (3·1%)
Any symptoms	301 (56·7%)	9,951 (37·0%)	48 (44·9%)	8,308 (28·4%)
Any TB related symptoms^*^	289 (54·4%)	9,178 (34·2%)	44 (41·1%)	7,027 (24·0%)

Numbers are count (%) unless otherwise specified.

Abbreviation: BMI-body mass index; DM-diabetes mellitus

^*^Cough, weight loss, night sweat, and fever.

### Model development

**Figure 1:**
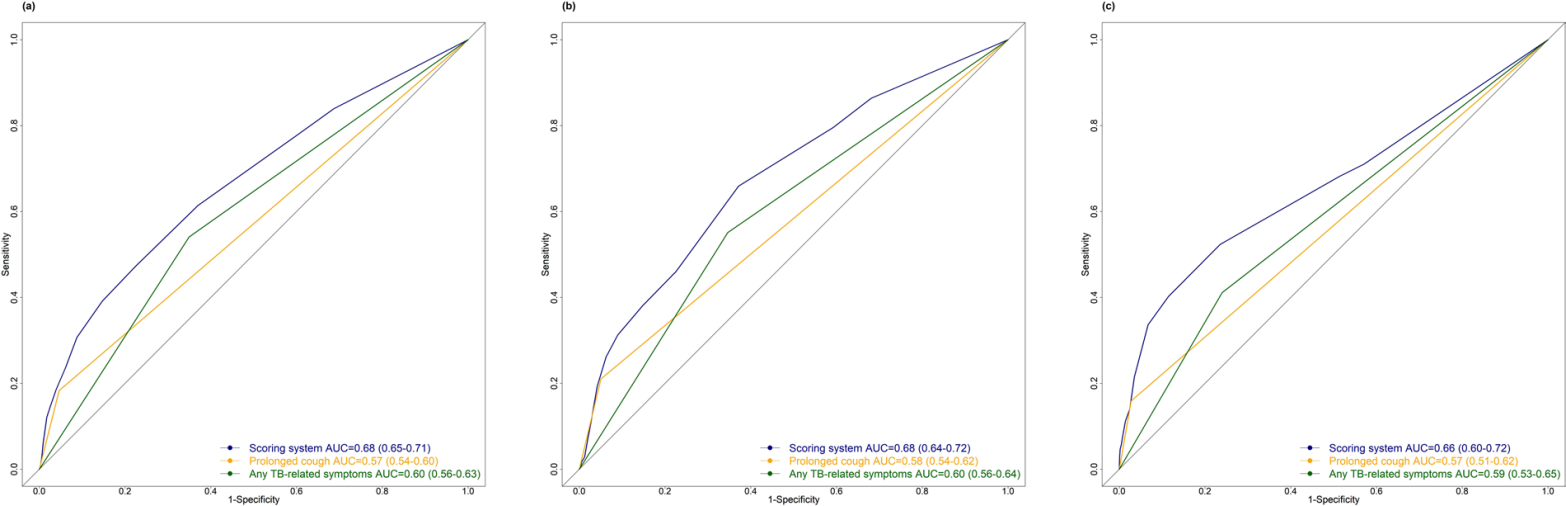
ROC Curve of screening options in different datasets. Performance of the screening strategies in South African training dataset (**a**), South African validation set (**b**) and Zambian dataset (**c**).

After backward variable selection, the final prediction model included the following variables: cough for less than two weeks, prolonged (> = 2 weeks) cough, night sweats, weight loss, known personal TB history, known household TB history, ever smoking, and ever using alcohol. In the final multivariable model, prolonged cough (OR 3·2, 95% CI 2·3–4·4) was the predictor with the highest OR (Table 2). The possible sum of scores in the scoring system ranged from 0 to 13. The AUC of the scoring system in SA training dataset was 68% (95% CI 65–71%), which was higher than that for prolonged cough (57%; 95% CI 54–60%) and any TB symptom (60%; 95% CI 56–63%) in the same dataset (Fig. 1a). The AUC of the scoring system (68%) was similar to that of the original logistic regression model (67%).

**Table 2:** Predictors and corresponding scores in the final selected prediction model based on the South African training dataset.

Predictor	aOR (95% CI)	Beta coefficient	Original score	Final score (rounded)
Weight loss	1·4 (1·1, 1·9)	0·35	1·458	1
Night sweats	1·5 (1·2, 2·0)	0·42	1·750	2
Cough <2 weeks	1·7 (1·3, 2·4)	0·56	2·333	2
Cough > = 2 weeks	3·2 (2·3, 4·4)	1·17	4·875	5
Ever drink	1·5 (1·2, 1·8)	0·38	1·583	2
Ever smoke	1·3 (1·0, 1·6)	0·24	1·000	1
Personal TB history	1·5 (1·1, 2·0)	0·41	1·708	2
Household TB history	1·4 (1·0, 1·9)	0·33	1·375	1

Abbreviation: CI-confidence interval; aOR-adjusted odds ratio.

### Internal and external validation

**Figure 2:**
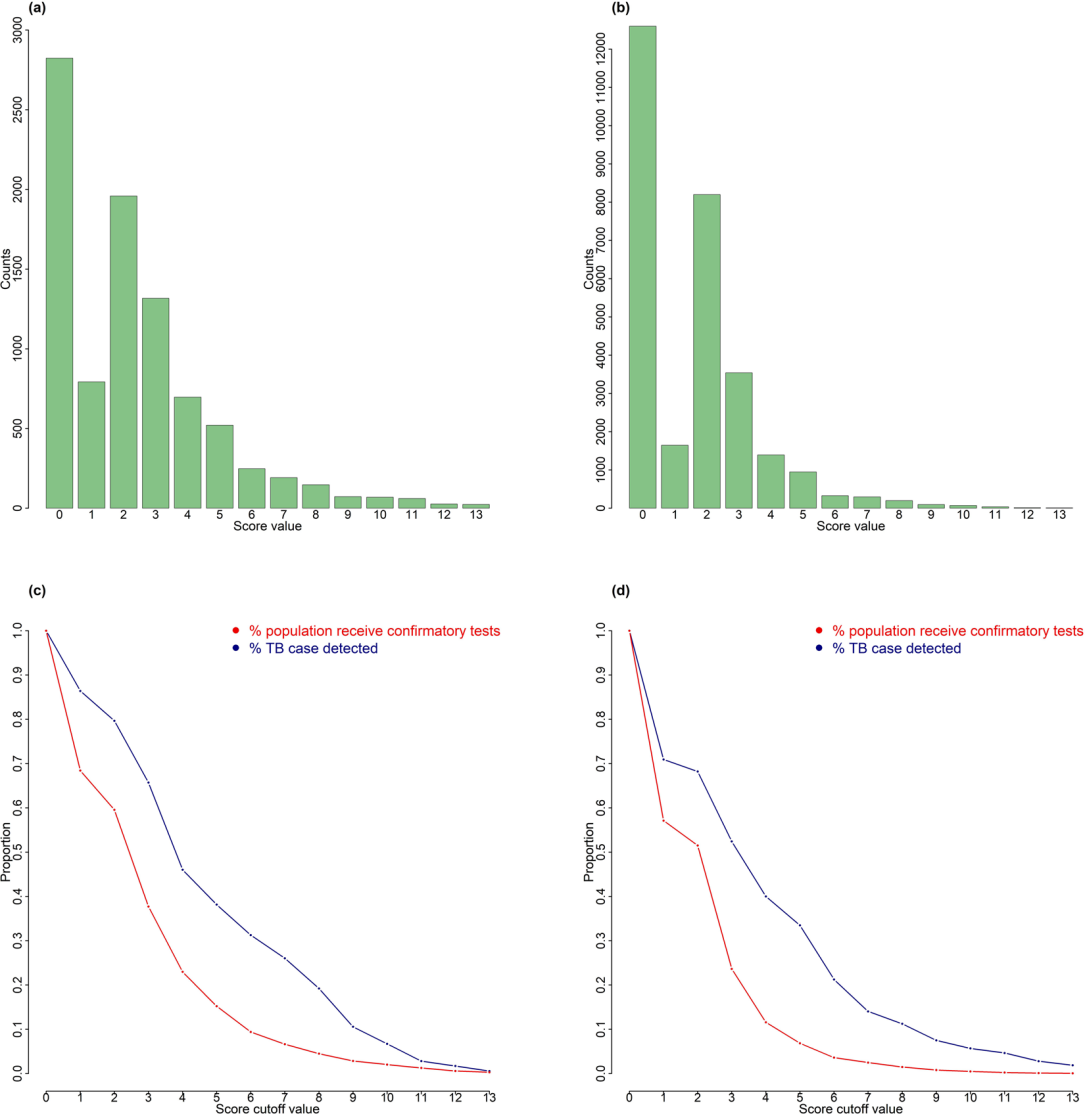
Performance of scoring system in validation datasets. Number of individuals at each score cutoff point in South African validation set (**a**) and Zambian dataset (**b**). Proportion of total screened population requiring further confirmatory tests at various cutoff values of score and proportion of all true TB cases detected at corresponding cutoff scores in South African validation set (**c**) and Zambian dataset (**d**).

In the SA (internal) validation dataset, the scoring system had an AUC of 68% (95% CI 64–72%), which was higher than prolonged cough (AUC 58%, 95% CI 54–62%) and any TB related symptom (AUC 60%, 95% CI 56–64%) (Fig. 1b). The AUC of the scoring system in the Zambian (external) dataset was 66% (95% CI 60–72%). The value was higher than that of prolonged cough (AUC 57%, 95% CI 51–62%) and any TB symptom (AUC 59%, 95% CI 53–65%) (Fig. 1c). The distribution of total score among the SA and Zambian validation populations is right skewed, with the majority of people having a score of less than 3 (Fig. 2a,b). The probability of prevalent TB increases with increasing score. In the SA validation dataset, the proportion of population who would require a confirmatory test increases rapidly when the score cutoff falls below six points (Fig. 2c). At a cutoff score of six points, 9·4% of the total population would require confirmatory testing and 31·3% of total TB cases would be detected eventually by this scoring system. Using a cutoff value of five points in the Zambian dataset, the screening algorithm which incorporated the scoring system could detect over 40% of all TB cases from 12% of the population who would screen positive (Fig. 2d).

### Cost-effectiveness analysis

**Figure 3:**
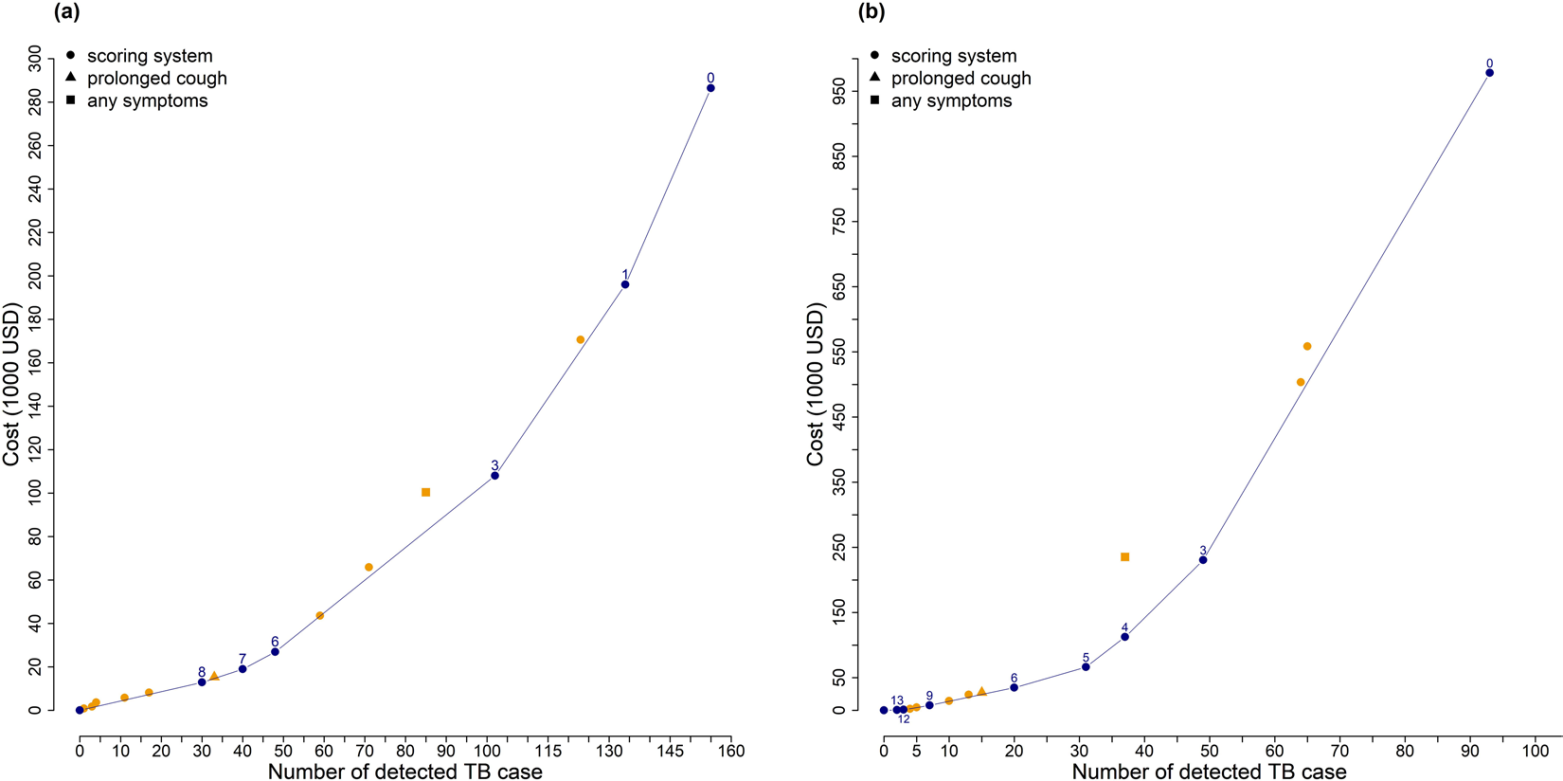
Cost-effectiveness plane. Cost-effectiveness analysis in South African validation dataset using the algorithm with Xpert MTB/RIF as the confirmatory tool (**a**). Cost-effectiveness analysis in Zambian dataset using the algorithm with smear plus Xpert MTB/RIF as the confirmatory tool (**b**). Orange dots indicated the dominated options.

In the SA (internal) validation dataset, a cost-effectiveness analysis was conducted for a systematic screening algorithm that used an initial screening method followed by Xpert MTB/RIF. With a decreasing cutoff value of the scoring system, the number of prevalent TB cases that could be detected increased, along with increased expenditure on systematic screening (Fig. 3a). The average cost per TB case detected (ACER) ranged from 429$USD to 1,848$USD under various cutoff values (Table 3). If the TB program is to maximize the number of TB cases detected given the budget, it should follow the right lower part of the cost effectiveness plane (i.e., the blue curve in Fig. 3a) depending on budget availability. It can be seen from the cost effectiveness plane that the conventional symptom-based strategies would be “dominated” by other options, since the TB program would never use them under any budget plan (Fig. 3a and Table 3). In the 1000 bootstrapped samples, the systematic screening algorithms using the scoring system dominated that using any TB symptom (99% of the samples) and prolonged cough (69% of the samples). Among the viable options (i.e., the blue curve in Fig. 3a), the cost per TB case detected increased if the program sets to find more TB cases (see Table 3 for the incremental rations comparing one option to the next viable option, or ICERs).

**Table 3:** Cost-effectiveness analysis in South African validation dataset (N = 8,952, TB prevalence = 1.97%) applying the diagnostic algorithm in Appendix Fig. 2(a), with Xpert MTB/RIF as the only confirmatory tool.

Score Cutoff	Number of detected TB case (95% CI)	Proportion of detected TB case (95% CI)	Cost USD (95% CI)	ACER (95% CI)	ICER^*^
13	1 (0, 3)	0·5 (0·0, 1·8)	768 (480, 1,088)	800 (237, -)	Dominated
12	3 (0, 6)	1·7 (0·0, 3·4)	1,632 (1,216, 2,112)	560 (282, -)	Dominated
11	4 (1, 9)	2·4 (0·6, 4·9)	3,584 (2,944, 4,320)	848 (410, 3,392)	Dominated
10	11 (4, 17)	6·0 (2·6, 9·6)	5,760 (4,992, 6,689)	544 (342, 1,217)	Dominated
9	17 (10, 25)	9·4 (5·7, 13·7)	8,096 (7,168, 9,088)	488 (330, 814)	Dominated
8	30 (19, 40)	16·9 (11·5, 22·2)	12,832 (11,552, 14,080)	429 (319, 637)	428
Prolonged cough	33 (23, 44)	18·4 (13·5, 24·1)	15,264 (13984, 16,672)	471 (354, 665)	Dominated
7	40 (28, 53)	22·9 (17·5, 28·7)	18,944 (17,503, 20,418)	472 (362, 664)	611
6	48 (35, 61)	27·5 (21·4, 33·3)	26,848 (25,183, 28,480)	557 (440, 752)	988
5	59 (46, 73)	33·5 (27·5, 39·5)	43,520 (41,471, 45,569)	734 (598, 945)	Dominated
4	71 (56, 88)	40·5 (34·1, 47·1)	65,792 (63,488, 68,288)	925 (759, 1,158)	Dominated
Any TB symptoms^**^	85 (70, 104)	48·6 (42·7, 55·3)	100,336 (97,566, 103,296)	1,179 (974, 1,445)	Dominated
3	102 (84, 120)	57·9 (51·7, 63·9)	108,000 (105,278, 110,720)	1,057 (897, 1,273)	1,503
2	123 (103, 143)	70·1 (64·8, 75·1)	170,592 (167,904, 173,441)	1,381 (1,189, 1,633)	Dominated
1	134 (112, 154)	76·0 (71·4, 80·1)	195,952 (193,407, 198,849)	1,460 (1,269, 1,753)	2,749
0	155 (133, 178)	88·0 (87·7, 88·3)	286,464 (286464, 286,464)	1,848 (1,609, 2,154)	4,310

Median of each screening method from the results of bootstrapping (1000 times) was shown with the confidence interval (CI).

Abbreviation: ACER-average cost-effectiveness ratio, ICER- Incremental cost-effectiveness ratio.

^*^Calculated based on the median values of the cost and TB cases detected from the 1000 bootstrapped samples.

^**^Cough, weight loss, night sweat, and fever.

In the Zambian (external) validation dataset, the systematic screening algorithm of initial screening followed by sequential confirmatory tests of smear microscopy plus Xpert MTB/RIF was evaluated (Appendix Fig. 2b). Similar to the SA situation, the new scoring system dominated the conventional strategies of any TB symptoms (100% of the bootstrapped samples) and prolonged cough (58% of the samples) in terms of cost-effectiveness (Fig. 3b). The ACERs and ICERs of the screening strategies (including the scoring system and the symptom-based strategies) in the Zambian cohort were substantially larger compared to those in the SA cohort, because of the lower TB prevalence (Table 4).

**Table 4:** Cost-effectiveness analysis in Zambian validation dataset (N = 29,381 TB prevalence = 0.36%) applying the diagnostic algorithm in Appendix Fig. 2(b), with sputum smear as the first confirmatory tool and Xpert MTB/RIF as the secondary confirmatory tool among smear-negatives.

Score Cutoff	Number of detected TB case (95% CI)	Proportion of detected TB case (%) (95% CI)	Cost USD (95% CI)	ACER (95% CI)	ICER^*^
13	2 (0, 4)	1·9 (0·0, 4·2)	342 (138, 548)	171 (64, -)	171
12	3 (0, 6)	2·6 (0·0, 5·8)	786 (510, 1094)	296 (131, -)	444
11	4 (1, 9)	3·7 (1·0, 8·2)	2,046 (1,574, 2,556)	512 (229, 2,044)	Dominated
10	5 (2, 10)	4·7 (1·8, 9·2)	4,366 (3,684, 5,150)	871 (430, 2,486)	Dominated
9	7 (3, 12)	6·5 (2·7, 11·1)	7,531 (6,684, 8,525)	1,061 (611, 2,827)	1,686
8	10 (5, 17)	9·6 (4·5, 15·5)	14,104 (12,818, 15,505)	1,387 (822, 2,848)	Dominated
7	13 (7, 20)	12·2 (7·1, 18·4)	23,897 (22,237, 25,675)	1,860 (1,181, 3,239)	Dominated
Prolonged cough	15 (8, 23)	13·6 (8·4, 20·4)	27,153 (25,377, 28,925)	1,860 (1,175, 3,217)	Dominated
6	20 (13, 29)	18·5 (12·5, 25·3)	34,649 (32,674, 36,661)	1,761 (1,195, 2,680)	2,086
5	31 (22, 42)	29·1 (21·4, 37·4)	66,191 (63,526, 68,838)	2,144 (1,566, 3,017)	2,867
4	37 (27, 49)	34·8 (26·7, 42·6)	112,484 (109,081, 116,033)	3,043 (2,326, 4,137)	7,715
Any TB symptoms^**^	37 (28, 50)	35·6 (27·7, 43·6)	235,155 (230,308, 239,459)	6,272 (4,700, 8,426)	Dominated
3	49 (37, 62)	45·5 (37·7, 53·2)	230,445 (225,577, 235,110)	4,730 (3,717, 6,251)	9,830
2	64 (50, 78)	59·4 (51·1, 66·7)	503,436 (498,203, 509,313)	7,931 (6,470, 10,154)	Dominated
1	65 (51, 81)	61·7 (54·1, 69·0)	558,333 (553,013, 563,972)	8,520 (6,901, 10,933)	Dominated
0	93 (77, 111)	87·0 (86·5, 87·5)	978,122 (977,769, 978,474)	10,518 (8,807, 12,707)	16,992

Median of each screening method from the results of bootstrapping (1000 times) was shown with the confidence interval (CI).

Abbreviation: ACER-average cost-effectiveness ratio.

^*^Calculated based on the median values of the cost and TB cases detected from the 1000 bootstrapped samples.

^**^Cough, weight loss, night sweat, and fever.

**Figure 4:**
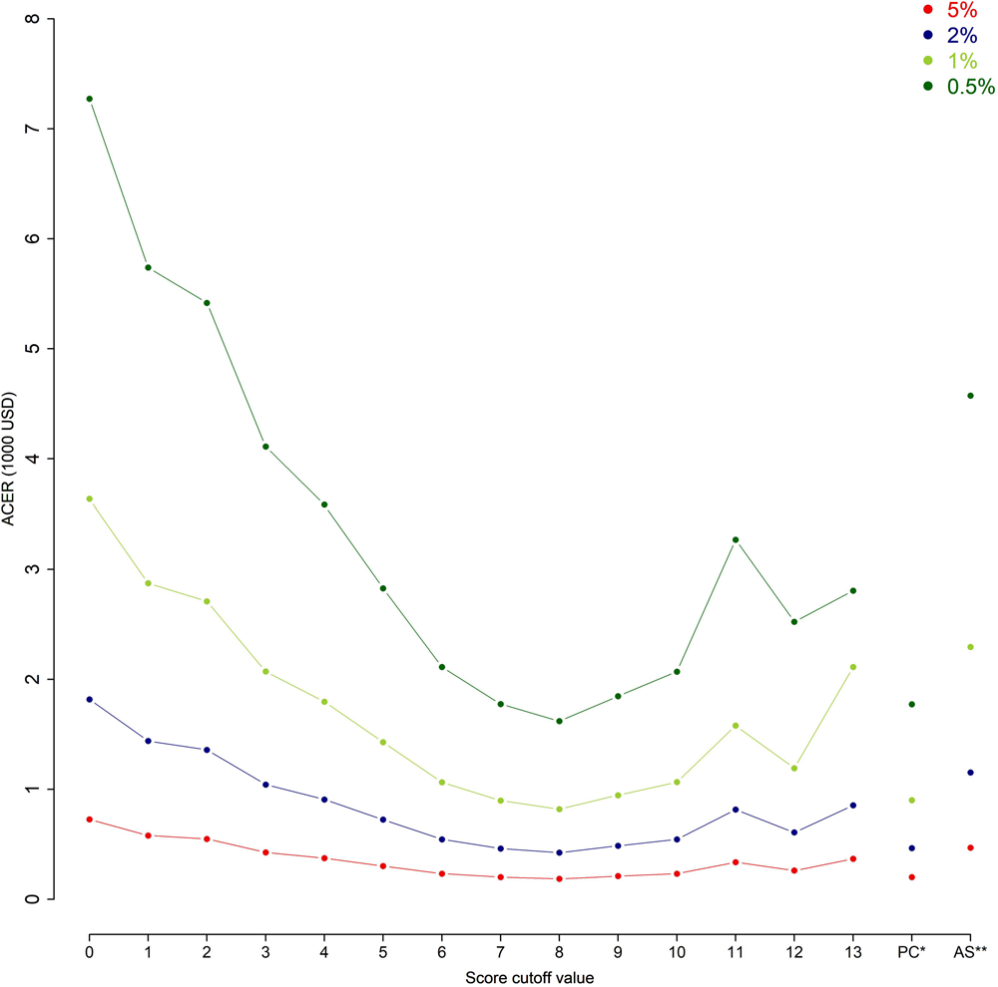
Cost-effectiveness analysis in hypothetical population under various background prevalence of TB. Average cost-effectiveness ratio (ACER) of diagnosis algorithm with scoring system as screening tool and Xpert MTB/RIF as confirmatory tool in different TB prevalence setting. Performance of the scoring system in South African validation set was shown. ^*^PC: Prolonged cough; ^**^AS: Any TB-related symptoms (Cough, weight loss, night sweat, and fever).

Lastly, the results of cost-effectiveness analysis in the hypothetical populations with different TB prevalence suggested that cost per TB case detected was highly affected by the background TB prevalence (Fig. 4). The ACERs were generally very high (>2,112$USD per TB case detected) when the background TB prevalence fell below 1%. When the background prevalence of TB was as high as 5%, the ACERs were generally below 800$USD and can be as low as 187$USD per TB case detected.

### Sensitivity analysis

In the sensitivity analysis, multiple imputation was used to impute missing predictor information (mainly for BMI) and the analysis repeated. Predictors selected included weight loss, night sweats, cough less than two weeks, prolonged cough, alcohol use, BMI, sex, known personal TB history, and known household history. In addition to weight loss, BMI was selected in the final model and had the highest score among those predictors (Appendix Table 2). Similar to the results in the main analysis, the scoring system from the sensitivity analysis had a higher AUC value than conventional screening tools in the SA and Zambian validation datasets (Appendix Fig. 3). The systematic screening algorithm using the new scoring system again dominated those that used the conventional tools in the cost-effectiveness analysis (Appendix Fig. 4).

## Discussion

To our knowledge, this study is the first to employ prevalence survey data to develop and validate a prediction model for prevalent TB among HIV negative or HIV status unknown individuals. The new scoring system that incorporated TB symptoms and risk factors were found to have better discriminatory power over conventional symptom-based screening strategies, although the overall discriminatory performance was only modest with an AUC of 66–68%. Importantly, the scoring system also performed better than the conventional strategies in terms of the number of TB cases detected given a fixed budget. Nonetheless, the tradeoff between sensitivity and specificity was still observed in the scoring system, and the cost per TB case detected remained relatively high unless the background TB prevalence was very high.

Previous discussions on systematic screening for TB using identification of symptoms as the first screening step have focused on the use of single or multiple TB symptoms as the rule-in criteria. Systematic reviews have concluded that these symptoms criteria would either miss a majority of TB cases in the population (because of low sensitivity) or lead to an overuse of confirmatory diagnostics among individuals without TB (because of low specificity)^4^. In addition, a recent systematic review identified only a few prediction models of prevalent TB and most of the studies were of poor quality and not externally validated^6^. One recently reported paper used the same ZAMSTAR dataset to develop potential screening strategies for prevalent TB. However, only various combinations of TB-related symptoms as the screening methods were considered^22^. Few studies have attempted to explore the potential of a multivariable modelling approach that incorporate additional risk factor information beyond TB symptoms and the magnitude of association between the predictors and TB. One prediction model of TB was developed previously using patients attending an outpatient clinic in Brazil, focusing on TB symptoms as predictors^23^. However, the predictors selected in the final model were not reported and the results were not externally validated.

Our study was among the very few TB prediction models that applied both internal validation in the same setting as the model development cohort and external validation in a setting with somewhat different TB and HIV epidemiology. The discriminatory performance of the prediction model was well preserved in the SA validation set (AUC: 68%, 64–72%), probably because the original training set had a large sample size and would not suffer from overfitting. The model performance was slightly decreased in the Zambian validation set (AUC: 66%, 60–72%), reflecting different epidemiology and clinical manifestations of TB in Zambia. Nonetheless, our modelling approach could still be applied to other settings when data are available. Using prolonged cough as an indicator to predict TB was previously reported to detect 35% (95% CI: 24–46%) of TB cases^5^, but in our validation sets, the sensitivity of prolonged cough was only 14·0% (Zambian dataset) and 18·8% (SA validation set), which indicates that the performance of the current symptom screening methods might differ in various settings.

In the absence of a single best cutoff that could achieve both high sensitivity and specificity, the key question of systematic screening will be the determination of an adequate cutoff under a budget constraint. Our cost-effectiveness analysis could help the TB program select the screening strategy, depending on the program’s budget and the willingness to pay to detect one additional TB case (the “unit price”). For example, under a total budget of up to 12,832$USD in our internal validation (SA) cohort, using a cutoff value of 8 would be the best option if the program accepts the price of 428$USD per TB case detected (Fig. 3a and Table 3). If the budget would be anywhere between 12,832$USD and 18,944$USD, one would first apply the confirmatory test to all subjects with a score of > = 8 points, and then apply the confirmatory test to subjects with a score of seven points until the budget is used up (Fig. 3a). Moving from the cutoff of 8 to 7 represents an incremental ratio (ICER) of 611$USD per TB case detected, meaning that the cost per TB case detected would increase if the program wants to find more TB cases.

Going beyond the simple dichotomy of risk stratification in conventional symptom-based strategies (e.g., prolonged cough or not), the scoring system provides a more flexible option of risk stratification through adopting different cutoff values. In addition, we found that the use of the scoring system would result in more TB cases detected compared to conventional strategies given the same budget, although the improvement was only modest. Nonetheless, our cost-effectiveness analysis revealed high ACERs and ICERs for both the scoring system and the symptom-based strategies when the screening was followed by confirmatory TB diagnostics. High costs for detecting a prevalent TB case present practical challenges. Previous studies have also showed that cost per TB case detected was high in community wide screening^24^. Our hypothetical analysis of applying the scoring system to populations with different TB prevalence suggested that the ACER would be high unless the systematic screening is applied to a high prevalence setting (2% or higher).

Our study has several strengths when considering its application to active case finding programs. First, the setting is similar to a community-based case-finding program. Second, cost-effectiveness analyses were conducted comparing diagnostic algorithms using different initial screening methods and different cutoff values of the scoring system which can help select the most feasible initial screening method in a screening program depending on budget constraints. Third, the model was validated not only in a SA dataset but also in a Zambian dataset, and the results suggested that the better discriminatory performance of the scoring system observed in the SA training dataset could not be explained by chance or overfitting.

The study also has limitations. First, we only included the cost of confirmatory diagnostics and did not consider the cost of staff time of conducting systematic screening and the patient cost, nor did we consider the possible intermediate screening step using chest radiography. Second, the outcome of the study was undiagnosed and bacteriologically confirmable prevalent pulmonary TB. Some of the prevalent cases detected in systematic screening might be detected anyway by passive case detection. We also did not account for the potential impact of systematic screening on transmission and incidence of TB. Third, the information in the scoring system was only based on self-report; therefore the performance of the prediction model may not be optimal. We argue that for the scoring system to be used easily in the community, the predictor information should be readily available with minimal further testing (e.g., blood glucose or HIV testing). Fourth, we only included in our prediction model the risk factors available in the ZAMSTAR study. Fifth, the study population was in Sub-Saharan Africa only; thus, the model might not be applicable in South-east Asia or other regions. Further studies in different areas with various TB epidemiology and healthcare settings are needed. Lastly, we did not include people living with HIV in the present analysis, since these people may have different clinical manifestations and risk factors of TB and should be evaluated separately. In practice, our screening algorithm on HIV negative and HIV unknown individuals can be combined with existing or upcoming screening algorithm on people living with HIV when being implemented in the community^7^.

## Conclusion

Using data from a large prevalence survey, we developed and validated the first scoring system to be used in systematic case finding among HIV negative or HIV unknown individuals. Although the scoring system dominated the conventional screening strategies by only a margin, it provided more flexible screening options to fill the shortcomings of current screening methods. Our scoring system and results from cost-effectiveness analysis can be used to inform targeted systematic case finding efforts in community settings with a similar TB and HIV epidemiology as in our study cohorts. Our study also provided a framework for future studies to follow and develop other prediction models based on locally available data. Depending on the available budget in TB screening programs, health care systems would be able to target high risk populations for diagnostic tests by choosing different cutoff values of the scoring system.

## Supplementary information

Appendix

## Data Availability

The study protocol did not state that data will be made publicly available and therefore open access to data has not been approved by the three ethics committees (at Stellenbosch University, the University of Zambia and the London School of Hygiene and Tropical Medicine). The data contain sensitive information such as HIV and TB status and participants did not give permission that this data can be made publicly available. Data can be made available to individual researchers who present an analysis plan to PIs and sign a data sharing agreement. Requests may be sent to mcla@sun.ac.za.
