# Supplementary material for: Development and validation of a prediction model for active tuberculosis case finding among HIV-negative/unknown populations
Source: Sci Rep. 2019 Apr 16;9:6143. doi: 10.1038/s41598-019-42372-x (PMC6467872; doi:10.1038/s41598-019-42372-x)
Supplement: Supplementary file 1 — Appendix [file 41598_2019_42372_MOESM1_ESM.pdf]

Development and validation of a prediction model for active tuberculosis case finding among HIV-negative/unknown populations

Authors

Yun-Ju Shih MSc[1], Helen Ayles PhD[2], Knut Lonnroth PhD[3], Mareli Claassens* PhD[4], Hsien-Ho Lin* ScD[1]

Affiliations

1. Institute of Epidemiology and Preventive Medicine, National Taiwan University, Taipei, Taiwan

2. Department of Clinical Research, London School of Hygiene and Tropical Medicine

3. Department of Public Health Sciences, Karolinska Institutet, Stockholm, Sweden

4. Desmond Tutu Tuberculosis Centre, Department of Paediatrics and Child Health, Stellenbosch University, Cape Town, South Africa

*Co-senior authors

Correspondence to: Dr. Hsien-Ho Lin, Institute of Epidemiology and Preventive Medicine, National Taiwan University, Taipei 100, Taiwan.

Email: hsienho@ntu.edu.tw

Appendix Table 1. Screening tools and confirmatory tools proposed in diagnosis algorithms.

Screening tool	Basic value
Scoring system	Sensitivity and specificity varies by different cutoff values in the scoring system
**Confirmatory Tool**	**Basic value**
Xpert MTB/RIF as the first confirmatory tool	Sensitivity: 88%, Specificity: 99%[1]<br>Cost: 32 USD[2]
Smear microscopy	Sensitivity: 61%, Specificity: 98%[3]<br>Cost: 2 USD[4]
Xpert MTB/RIF as the second confirmatory tool (among smear-negative individuals)	Sensitivity: 67%, Specificity: 98%[1]<br>Cost: 32 USD[2]

Performance of the tools and cost were used in the cost effectiveness analysis. *Cough, weight loss, night sweat, and fever.

1. Steingart KR, Schiller I, Horne DJ, Pai M, Boehme CC, Dendukuri N. Xpert(R) MTB/RIF assay for pulmonary tuberculosis and rifampicin resistance in adults. Cochrane Database Syst Rev. 2014(1):CD009593.
2. Meyer-Rath G, Schnippel K, Long L, MacLeod W, Sanne I, Stevens W, et al. The impact and cost of scaling up GeneXpert MTB/RIF in South Africa. PLoS One. 2012;7(5):e36966.
3. Steingart KR, Henry M, Ng V, Hopewell PC, Ramsay A, Cunningham J, et al. Fluorescence versus conventional sputum smear microscopy for tuberculosis: a systematic review. Lancet Infect Dis. 2006;6(9):570-81.
4. Lu C, Liu Q, Sarma A, Fitzpatrick C, Falzon D, Mitnick CD. A systematic review of reported cost for smear and culture tests during multidrug-resistant tuberculosis treatment. PLoS One. 2013;8(2):e56074.

Appendix Table 2. Predictors and corresponding scores of the prediction model.

Predictor	Unadjusted OR (95% CI)	aOR (95% CI)	Beta coefficient	Score
Weight loss	2.4 (1.8, 3.0)	1.3 (1.0, 1.7)	0.24	1
Night sweats	2.5 (2.0, 3.1)	1.6 (1.2, 2.0)	0.44	2
Cough <2 weeks	2.3 (1.6, 3.1)	1.8 (1.3, 2.5)	0.59	2
Cough >=2 weeks	5.0 (3.8, 6.7)	3.3 (2.4, 4.6)	1.21	5
Ever drink	1.9 (1.5, 2.4)	1.4 (1.1, 1.8)	0.33	1
Women	0.7 (0.6, 0.9)	1.3 (1.0, 1.7)	0.25	1
Personal TB history	2.1 (1.6, 2.7)	1.3 (1.0, 1.8)	0.26	1
Household TB history	1.3 (1.0, 1.8)	1.3 (1.0, 1.8)	0.27	1
Normal weight	2.5 (1.8, 3.4)	2.4 (1.6, 3.5)	0.88	4
Under weight	5.4 (2.9, 10.1)	4.5 (2.2, 9.6)	1.51	6

Using multiple imputation to impute missing predictor information. Results were pooled from 10-fold multiple imputation using the Rubin's rule. Abbreviation: CI-confidence interval; OR-odds ratio; aOR-adjusted odds ratio

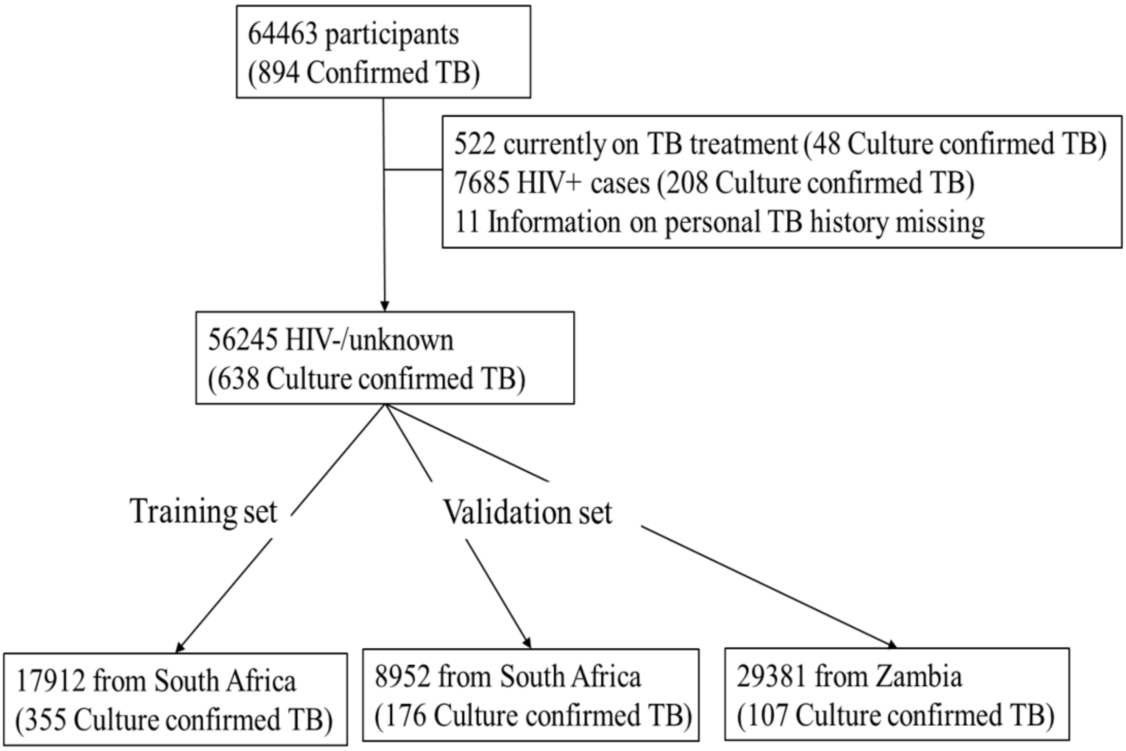

Appendix Figure 1. Flow diagram of the study participants.

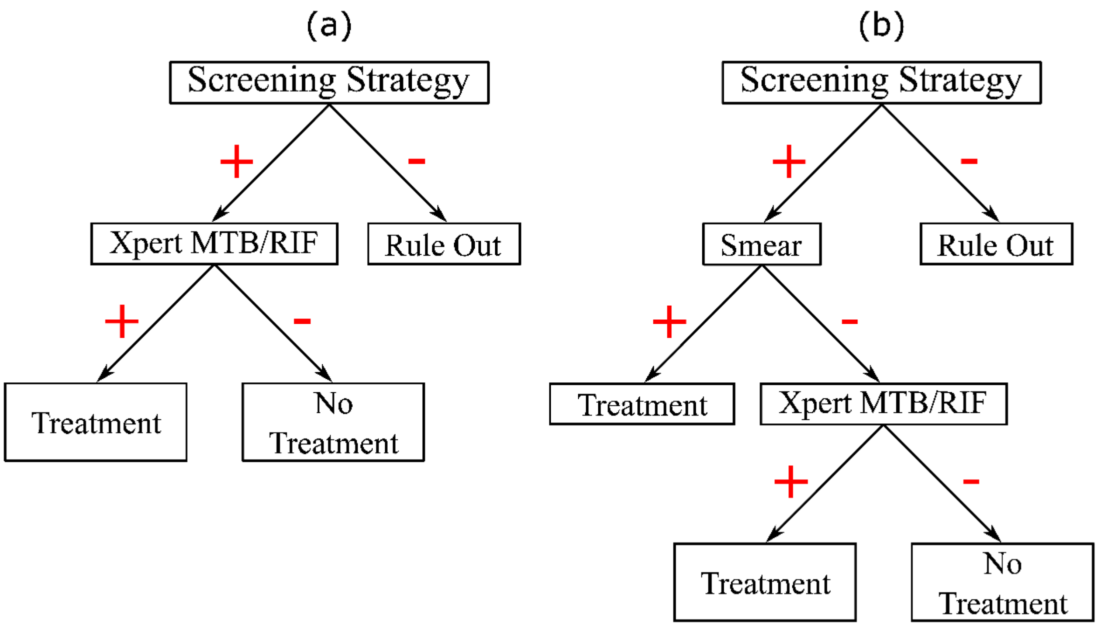

Appendix Figure 2. Diagnosis algorithms for cost-effectiveness analysis. First algorithm was implemented in South African validation set (a). Second algorithm was used in Zambian data (b).

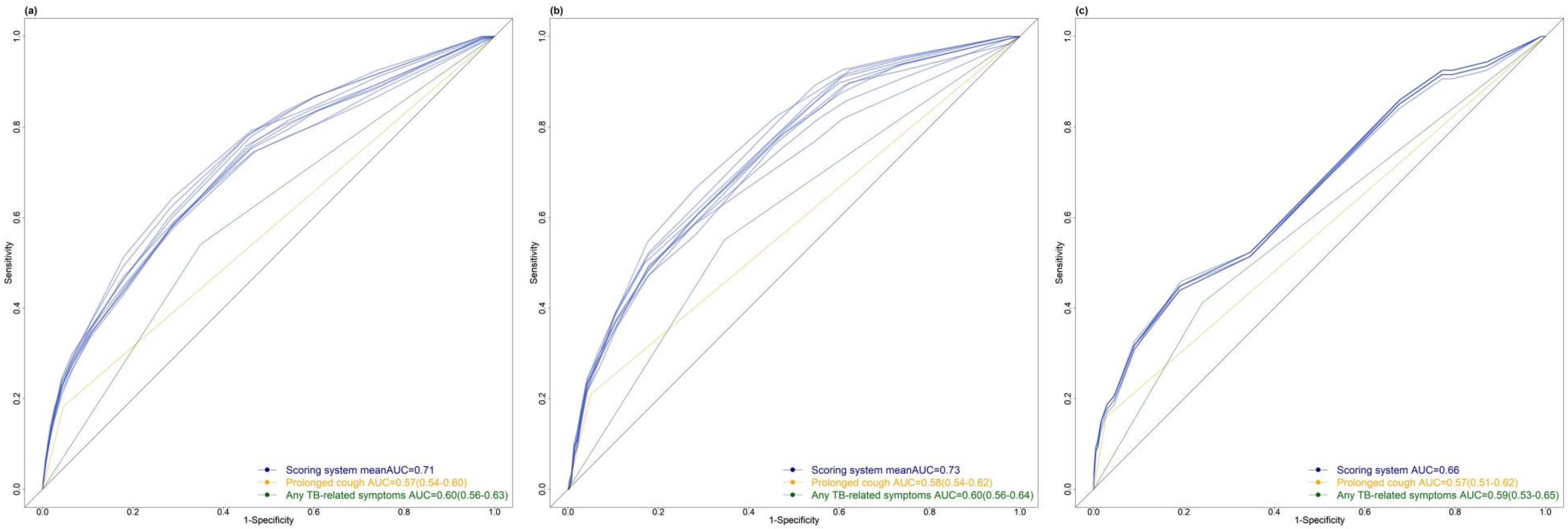

Appendix Figure 3. ROC curves of screening strategies in different datasets using multiple imputation. Performance of the screening strategies in South Africa training dataset (a), South Africa validation set (b) and Zambia dataset (c). Results on the new scoring system were from 10-fold multiple imputations.

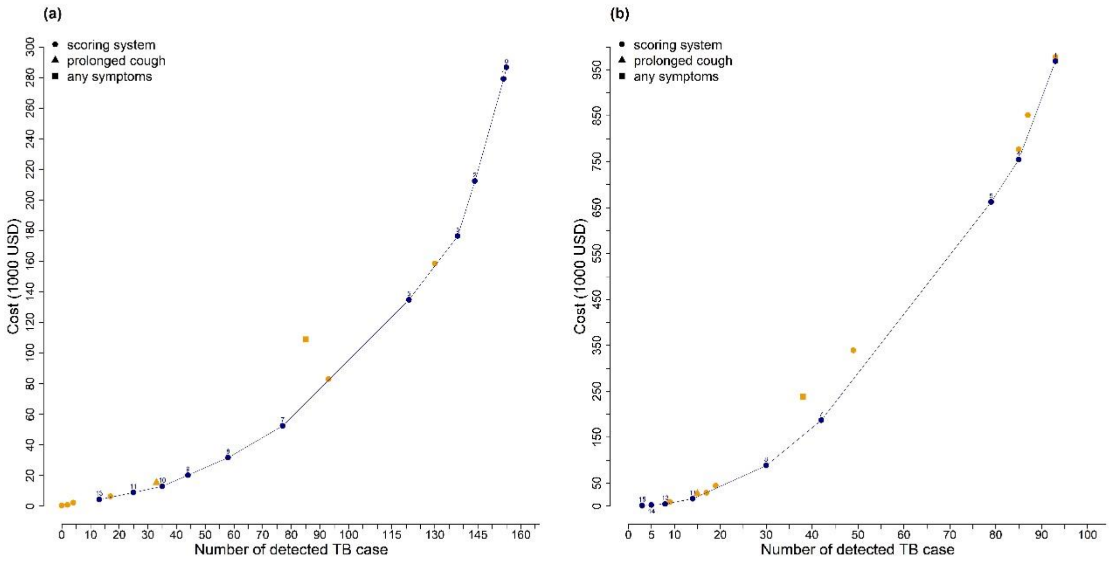

Appendix Figure 4. Cost-effectiveness plane of imputed SA validation dataset using the algorithm with Xpert MTB/RIF as the confirmatory tool (a). Cost-effectiveness plane of imputed Zambia datasets using the algorithm with smear microscopy plus Xpert MTB/RIF as the confirmatory tool (b). Orange dots indicated the dominated options.
